# The effect of sleep-oriented non-pharmacological interventions in preventing delirium: a systematic review of randomized controlled trials

**DOI:** 10.3325/cmj.2025.66.289

**Published:** 2025-08

**Authors:** Ezgi̇ Mutluay Yayla, Emi̇ne Kaplan Seri̇n

**Affiliations:** 1 Nursing Department, Health Sciences Faculty, Tarsus University, Tarsus, Turkey; 2 Department of Medical Nursing, Faculty of Nursing, Mersin University, Mersin, Turkey

## Abstract

**Aim:**

To systematically review randomized controlled trials evaluating the efficacy of sleep-oriented non-pharmacological interventions for delirium prevention in intensive care units.

**Methods:**

We searched PubMed, Web of Science, Scopus, and the Cochrane Library for randomized controlled trials evaluating the efficacy of sleep-oriented, non-pharmacological interventions for delirium prevention in intensive care units published in English between 2019 and 2024. The methodological quality of the included studies was independently evaluated by two researchers using the Joanna Briggs Institute tool.

**Results:**

This review included eight randomized controlled trials (n = 649). Six studies assessed the effect of earplugs and masks on delirium prevention in intensive care patients. Three studies indicated that earplugs and eye masks effectively prevented delirium, while three studies found no effect. One study assessed the use of earplugs and masks as part of a bundle of interventions and reported a positive effect. Four studies showed an effect on sleep. None of the sleep-oriented non-pharmacological interventions assessed in these studies reported any harmful effects.

**Conclusion:**

Although several high-quality studies showed that these interventions significantly lowered delirium incidence, some trials found no effect, highlighting variability in outcomes.

PROSPERO registration number: CRD42025642734

Delirium is a transient organic mental syndrome marked by a sudden onset, fluctuating symptoms, cognitive impairment, altered consciousness, and attention deficits, along with disruptions in psychomotor activity and the sleep-wake cycle (1,2). Delirium affects 20%-50% of critically ill, non-ventilated intensive care unit (ICU) patients, 50%-80% of mechanically ventilated patients, and reaches a cumulative incidence of 85% in end-of-life ICU care (3-5).

Delirium negatively affects patient outcomes, independently correlating with increased mortality, extended hospital stays, higher health care costs, prolonged mechanical ventilation, and elevated rehospitalization risk (6,7). Its precise etiology remains unclear, but it is believed to result from a confluence of multiple contributing factors. The risk factors and underlying causes of delirium are multifactorial, involving withdrawal states, acute metabolic abnormalities, traumatic injuries, central nervous system diseases, vitamin or mineral deficiencies, hypoxia, acute vascular events, endocrine dysfunction, and toxic exposures, such as drugs or heavy metals (8,9). The National Institute for Health and Care Excellence guidelines recommend that patients with risk factors for delirium are assessed for delirium within 24 hours of admission. Consequently, nurses' ability to recognize and diagnose delirium is essential for prompt intervention (10,11).

Although pharmacological interventions play a role in delirium management, they may not adequately prevent its onset. Consequently, primary prevention strategies emphasize non-pharmacological approaches, which encompass a range of interventions (12). These interventions target cognitive and perceptual disturbances (eg, disordered thinking and disorientation), physical risks (eg, immobility and falls), physiological factors (eg, sleep disruption and dehydration), and sensory and environmental concerns (8).

Sleep promotion has been a primary focus of non-pharmacological interventions for delirium management. Increasing sleep duration and improving sleep quality may contribute to delirium prevention. Furthermore, as sleep disturbance is a recognized symptom of delirium, the emphasis on sleep-related interventions is justified by the considerable overlap in symptom presentation (13-15).

Sleep disruption is strongly associated with delirium, and improving the physical environment to extend sleep duration and enhance sleep quality can reduce delirium risk (16). Reported effective non-pharmacological interventions for sleep promotion in delirium prevention encompass earplugs, eye masks, massage, relaxation therapies, foot baths, music interventions, targeted nursing protocols, acupressure, and aromatherapy. The aim of this study is to systematically review randomized controlled trials evaluating the efficacy of sleep-oriented non-pharmacological interventions for delirium prevention in ICUs over the past 5 years.

## METHODS

### Design

The review protocol was registered in PROSPERO (CRD42024548456), an international database for systematic review registration. The review adhered to the PRISMA-P guidelines. Two researchers independently conducted literature searches, selected articles, extracted data, and assessed the quality of the included studies.

### Search strategy

PubMed, Web of Science, Scopus, and the Cochrane Library were searched from December 2024 to February 2025. Search strategies incorporated controlled vocabularies, including Medical Subject Headings (MeSH), where applicable. The search terms were as follows: “delirium,” “non-pharmacological,” “sleep,” “intensive care,” and “randomized controlled trial.”

### Inclusion criteria

The selection of studies was guided by the PICOS (*P* = Population, I = Implementation, C = Comparison group, O = Outcome, S = Study design) framework. The criteria were as follows:

P: Intensive care patients aged 18 years and older

I: Patients undergoing sleep-oriented nonpharmacological intervention

C: Standard care

O: Studies reporting on sleep, delirium, or both combined

S: Randomized controlled trials published in English

This study included randomized controlled trials published in English between 2019 and 2024 focusing on non-pharmacological interventions in adult hospital patients. Only studies with accessible full texts were included.

In all the studies included in the analysis, daily delirium assessment was performed with validated tools like the Confusion Assessment Method (CAM), the Neelon and Champagne Confusion Scale (NEECHAM), the Intensive Care Delirium Screening Checklist (ICDSC), or a psychiatrist's evaluation based on the Diagnostic and Statistical Manual of Mental Disorders (DSM). Sleep quality was evaluated through subjective measures, such as validated sleep questionnaires: the Richard-Campbell Sleep Questionnaire (RCSQ), Verran Snyder Halpern Sleep Scale (VSHSS), and Pittsburgh Sleep Quality Index (PSQI). RCSQ is a five-item questionnaire that assesses patients’ perception of sleep on a visual scale from 0 to 100. The domains evaluated are depth of sleep, ease of falling asleep, frequency of waking up, ease of returning to sleep, and subjective sleep quality. VSHSS is a 15-item tool that evaluates subjective sleep quality, with higher scores on the sleep disturbance and sleep supplementation subscales indicating worse sleep, and higher scores on the sleep effectiveness subscale indicating better sleep. The PSQI consists of 19 individual questions that create seven component scores. It is a self-rated questionnaire used to measure sleep quality over the past month, with a score of 5 or more indicating poor sleep (12-16).

### Data extraction and analysis

Overall, 739 studies were retrieved from the databases. Duplicate articles were removed, and titles and abstracts were screened to exclude irrelevant studies. The remaining articles were assessed against the inclusion criteria. Full-text reviews were conducted as needed to confirm eligibility. Ultimately, 8 studies met the inclusion criteria and were included in the review.

Initially, the titles and abstracts were scanned, and then the 8 articles included in the review were examined in detail. Discrepancies between the two researchers were resolved through consensus. A standardized data extraction form, developed by the authors, was used to summarize study information, including the primary author, publication year, country of origin, study setting, sample size, intervention details, frequency and duration of delirium and sleep quality assessments, results, recommendations, and quality assessment (Table 1).

**Table 1:** The characteristics of the studies included in the review

Authors/y of study/place of study	Participants	Intervention group	Control group			Outcome assessed	Assessment tools for sleep measurement	Assessment tools for delirium	Outcomes measure	Conclusion	Joanna Briggs Institute scale
Arttawejkul et al /2020/ Thailand (17)	Patients admitted to medical intensive care unit (ICU) N = 17	N = 8 Six male (75%) and two female (25%) Mean age: 67 (18) Earplugs and eye masks: every night at 10 pm to 7 am	N = 9 Five male (56%) and four female (44%) Mean age: 76 (32) Usual routine			Sleep quality and prevalence of delirium	Pittsburgh Sleep Quality Index Questionnaire Richard-Campbell Sleep Questionnaire Verran/Snyder-Halpern Sleep Scale	Confusion Assessment Method for the ICU	There was no significant difference in the RCSQ scores and the prevalence of delirium between the two groups.	The two groups did not differ in the prevalence of delirium.	11
Akpinar, et al /2021/ Turkey (19)	Patients admitted to the coronary ICU N = 84	N = 42 24 male (57.1%) and 18 female (42.9%) Mean age: 64.04 ± 8.64 Earplugs and eye mask: from 22:30 pm to 6:30 am for 2 d	N = 42 22 male (52.4%) and 20 female (47.6%) Mean age: 63.73 ± 6.92 Usual routine			Sleep quality and the degree of delirium	Richards-Campbell Sleep Questionnaire	The Intensive Care Delirium Screening Checklist	The average RCSQ scores showed a significant difference between the groups when comparing second and third nights (*P* < 0.001). For the ICDSC scores, there was no significant difference between the experimental and control groups on the second night (*P* = 0.176), but there was a significant difference on the third night (*P* = 0.004).	Overnight use of earplugs and eye masks was linked to enhanced sleep quality and a decrease in the severity of delirium.	12
Faustino et al /2022/ Brazil (20)	Patients admitted to ICU N = 144	N = 72 36 male (50%) and 36 female (50%) Mean age: 68.5 (62.0–78.0) Five combined non-pharmacological interventions: sensory stimulation, reorientation, cognitive stimulation, environmental management, and the use of eye masks and earplugs.		N = 72 31 male (43.1%) and 41 female (56.9%) Mean age: 64.5 (57.0–75.5) Usual routine	The incidence density of delirium		-	Confusion Assessment Method for the ICU Richmond Agitation- Sedation Scale	No cases of delirium occurred in patients who used sleep promotion measures, hearing aids, photographs, bedside radios, and engaged in reading books and magazines of general interest.	Compared to standard care, the implementation of a bundle of non-pharmacological measures significantly reduced the incidence of delirium among critically ill patients.	11
Fazlollah et al /2021/ Iran (21)	Patients undergoing coronary artery bypass grafting N = 60	N = 30 18 male (60%) and 12 female (40%) Mean age: 63.43 ± 7.22 In the intervention group, foot reflexology massage was performed on each foot for 15 min, for two consecutive days.		N = 30 11 male (36.7%) and 19 female (63.3%) Mean age: 65.16 ± 7.09 Usual routine	Incidence of delirium, sleep quality, and pain intensity		Richard Campbell sleep questionnaire	Richmond Agitation- Sedation Scale Delirium Observation Screening Scale (version 0–1)	On the first day, delirium was observed in 10 patients in both groups, a non-significant difference. By the second day, 8 patients (26.7%) in the intervention group and 7 patients (23.3%) in the control group experienced delirium. This difference remained non significant (*P* = 0.76).	Foot reflexology did not effectively reduce delirium.	10
Kiliç and Kav/2023/ Turkey (22)	Patients admitted to ICU N = 60	N = 30 20 male (66.7%) and 10 female (33.3%) Mean age: 63.43 ± 17.91 Eye masks and earplugs: approximately 23:00 to 07:00 for 3 d		N = 30 20 male (66.7%) and 10 female (33.3%) Mean age: 66.87 ± 18.24 Usual routine	Delirium and sleep quality		Richard–Campbell Sleep questionnaire	Nursing Delirium Screening Scale Richmond Agitation- Sedation Scale	The intervention and control groups significantly differed in the development of delirium on the night of the 2nd day (*P* = 0.019), the day of the 3rd day (*P* < 0.001), and the night of the 3rd day (*P* ≤ 0.001). The average total sleep quality score was significantly higher in the intervention group across all three nights (*P* ≤ 0.001).	The use of earplugs and eye masks overnight effectively prevented delirium in intensive care patients.	11
Leong, et al /2021/Singapore (23)	Patients undergoing major abdominal surgery N = 93	N = 48 25 male and 23 female Mean age: 67 Earplugs and eye masks: 22.00 to 06.00 for 3 d		N = 45 26 male and 19 female Mean age: 60 Usual routine	Sleep quality, patient satisfaction, incidence of delirium		Richards-Campbell Sleep Questionnaire Pittsburgh Sleep Quality Index	Neelon and Champagne Confusion Scale	There was no significant difference between the groups in the efficacy of earplugs and eye masks to improve sleep quality and reduce the overall incidence of delirium over three postoperative days.	The use of earplugs and eye masks did not contribute to the development of delirium.	11

### Evaluation of methodological quality

The first and second authors independently assessed the methodological quality of the included studies. Discrepancies in their evaluations were resolved through consensus. The Joanna Briggs Institute (JBI) checklist for randomized controlled trials was used (25) (Table 1). The JBI quality assessment tools and checklists were validated for the Turkish language, with a moderate Cronbach's alpha coefficient (26). This 13-item checklist includes response options of yes (1), no (0), and unclear/not applicable (0). Higher total scores indicate higher methodological quality. Study quality assessments in this review ranged from 9 to 12.

## RESULTS

### Selection of studies

**Figure 1:**
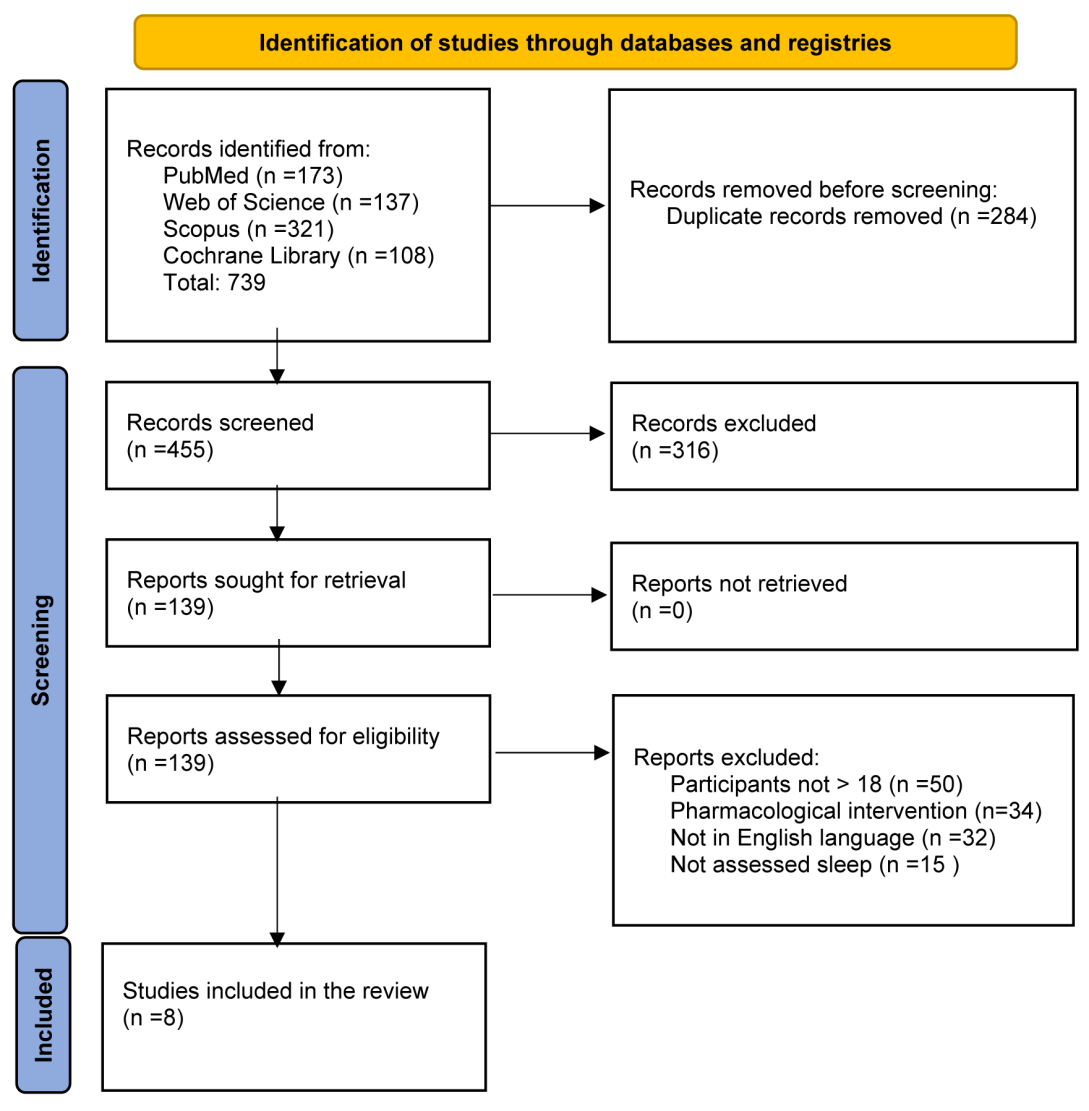
PRISMA 2020 study flow diagram.

The literature search initially retrieved 739 citations. A total of 284 duplicates were removed, leaving 455 items. Screening of titles and abstracts eliminated 316 irrelevant studies. A further 131 studies were eliminated during full-text review because they did not meet the inclusion criteria. This process resulted in the identification of 8 studies finally included in the systematic review (Figure 1).

### General characteristics of the studies and participants

The sample sizes ranged from 17 to 144, with a total of 649 participants. The intervention groups comprised 331 participants (166 male) and the control groups comprised 318 (156 male) (Table 1).

The included studies primarily focused on older adults in medical, surgical, and cardiac ICUs (17-24). One study evaluated a five-component non-pharmacological intervention bundle that included sleep promotion (20). One study examined the impact of foot reflexology massage on delirium incidence and sleep quality (21). Six studies assessed the efficacy of earplugs and eye masks for sleep protection in reducing delirium (17-19,22-24). Delirium was assessed in all the included studies using validated tools. Two studies used CAM-ICU (17,18), one used CAM-ICU and RASS (20), one used ICDSC (19), one used RASS and DOS Scale (21), one used Nu-DESC and RASS (22), one used only NEECHAM (23), and one used CAM-ICU and NEECHAM (24). Sleep quality was evaluated using sleep tools in seven studies. Four studies used RCSQ (18,19,21,22), one used PSQI, RCSQ, and VSHSS (17); one used VSHSS (24), and one used RCSQ and PSQI (23) (Table 1).

### Systematic review of outcomes: sleep quality and delirium

Arttawejkul et al (17) examined the effect of earplugs and eye masks on delirium prevalence in 17 medical ICU (MICU) patients from a single center. Delirium was assessed using the CAM-ICU scale for five days, while sleep was assessed using PSQI, RCSQ, and VSHSS. The groups significantly differed in neither delirium prevalence (*P* = 1.000) nor RCSQ scores (*P* = 0.236) (17).

Akpinar et al (19) examined 84 coronary ICU patients. The intervention group (n = 42) was encouraged to wear earplugs and eye masks overnight on their second and third nights of admission. Delirium severity was measured using the ICDSC, and sleep quality was assessed using the RCSQ. For the ICDSC scores, there was no significant difference between the experimental and control groups on the second night (*P* = 0.176), but there was a significant difference on the third night (*P* = 0.004). The comparisons between the scores on the second and third night revealed a significant difference in both RCSQ (χ^2^ = 70.393, *P* < 0.001) and ICDSC (χ^2^ = 10.906, *P* = 0.004) scores in the intervention group. However, no significant differences were observed in the control group (RCSQ: χ^2^ = 4.732, *P* = 0.09; ICDSC: χ^2^ = 1.542, *P* = 0.46) (19).

Faustino et al (20) assessed the effect of a multifaceted non-pharmacological intervention on delirium prevalence in 144 ICU patients from three centers. The intervention consisted of periodic reorientation, sensory deficit correction, environmental management, cognitive stimulation, and sleep promotion. Delirium was assessed twice daily using the CAM-ICU, RASS, and NEECHAM scales; however, sleep was not assessed. The intervention group showed a reduced incidence density of delirium (1.3 × 10^-2^ per person-days) compared with the control group (2.3 × 10^-2^ per person-days). Interestingly, no delirium cases occurred in patients receiving sleep promotion, bedside radios, photographs, hearing aids, or reading materials (20).

Fazlollah et al (21) evaluated the effect of foot reflexology massage on delirium incidence and sleep quality in 60 cardiac surgery patients. Foot reflexology massage was administered for 15 minutes to each foot for two consecutive days. Delirium incidence was measured using the RASS and DOS scale version across three shifts: morning, evening, and night. Sleep quality was assessed using the RCSQ. No significant difference in delirium incidence or RCSQ scores was observed between the intervention and control group (21).

Kiliç and Kav (22) examined the effect of nightly eye mask and earplug use on delirium prevention over three days in medical and surgical ICUs of a tertiary hospital. Delirium was evaluated with the Nu-DESC and RASS scales, while sleep quality was assessed with the RCSQ. Delirium incidence was significantly higher in the intervention group on the second night (*P* = 0.019) and during the third day (both day and night, *P* ≤ 0.001). Furthermore, the intervention group demonstrated a significantly higher average total sleep quality score across all three nights (*P* ≤ 0.001) (22).

Leong et al (23) enrolled 93 patients undergoing major abdominal surgery. The intervention group (n = 48) was encouraged to wear earplugs and eye masks on postoperative days 1-3. Delirium incidence was measured using the NEECHAM scale, and sleep quality was assessed using the RCSQ. No significant difference in overall delirium incidence or sleep quality improvement was observed between the two groups over the three postoperative days (23).

Obanor et al (18) evaluated the impact of earplugs and eye masks on sleep quality in 87 postoperative surgical ICU patients. Delirium was assessed using the CAM-ICU, and sleep quality was measured using the RCSQ. No delirium was detected in either group (all CAM-ICU scores were negative). However, the average RCSQ score in the intervention group (64.5; 95% CI 58.3-70.7) was significantly higher than that in the control group (47.3; 95% CI 40.8-53.8; *P* = 0.0007) (18).

Shorofi et al (24) investigated the impact of three consecutive nights of eye mask and earplug use on delirium severity and sleep quality in 114 coronary artery bypass grafting patients in a cardiac ICU starting on the second night post-admission. Delirium severity was measured with the NEECHAM scale, and sleep quality was assessed with the Verran and Snyder-Halpern Sleep Scale. Following the intervention, a significant difference in the mean delirium severity score was observed between the two groups on the second, third, and fourth postoperative days (*P* < 0.001) (24). Moreover, the intervention reported significantly higher scores on sleep quality domains throughout the three intervention days

### Evaluation of methodological quality

Most trials demonstrated strong methodological rigor, with appropriate randomization, balanced baseline characteristics, consistent treatment across groups, reliable outcome measurement, and proper statistical analysis. However, allocation concealment and blinding of participants, providers, and outcome reviewers were inconsistently applied, representing the main sources of potential bias. Overall, the majority of studies scored highly (10-12 points), indicating generally good quality, while one study (9 points) showed comparatively weaker methodological safeguards.

## DISCUSSION

This systematic review examined the effectiveness of sleep-oriented non-pharmacological interventions in preventing delirium among ICU patients. Current best evidence and consensus statements advocate for a non-pharmacological approach to delirium management, emphasizing prevention and early recognition. While pharmacological methods are often used to address patient sleep disturbances, non-pharmacological strategies also play a crucial role in preventing or mitigating these issues. Given delirium's multifactorial etiology, clinical guidelines from the Society of Critical Care Medicine recommend combined non-pharmacological interventions to prevent delirium and shorten its duration (27-29). Sound and light are two key modifiable environmental factors that contribute to sleep deprivation in patients. Literature supports the use of environmental modifications to promote sleep and reduce delirium risk (24,27,28). Reflexology is another non-pharmacological approach used in delirium management, with studies demonstrating its effectiveness in improving sleep quality (30,31).

Randomized controlled trials by Kılıç and Kav, Shorofi et al, and Akpinar et al demonstrated that eye masks and earplugs effectively prevented delirium in ICU patients (19,22,24). Similarly, a meta-analysis by Litton et al concluded that earplug use reduced delirium incidence (32). These findings align with the results of an observational study by Tonna et al (27) and a randomized controlled trial by van Rimpaey from 2012 (33). Faustino et al reported that the implementation of a non-pharmacological intervention bundle significantly reduced delirium incidence among critically ill patients (20). Patel et al in 2014 also demonstrated a significant decrease in delirium incidence using a non-pharmacological bundle focused on environmental noise and light reduction to minimize nighttime disturbances (34). According to a systematic review by Chen et al, the most effective strategy for preventing delirium in ICUs was a multicomponent intervention involving physical activity, family participation, cognitive stimulation, reorientation, sensory stimulation, environmental control, and clinical adjustment (35). Kang et al reported that multi-component interventions effectively reduced delirium incidence but not its duration (36). Conversely, Deng et al found that these interventions reduced both the duration of delirium and ICU stay, though their findings were not significant (37).

In contrast, randomized controlled trials by Arttawejkul et al, Leong et al, and Obanor et al found no reduction in delirium incidence with nightly earplug and eye mask use in ICU patients (17,18,23). Similarly, Fazlollah et al found reflexology to be ineffective in reducing delirium or improving sleep quality (21). However, other studies found foot reflexology massage a safe complementary medicine treatment used for delirium (38).

### Study limitations

The interpretation of this systematic review should consider several limitations. Our search was restricted to English-language publications, which may have excluded relevant studies in other languages. Additionally, our literature search was confined to the PubMed, Cochrane Library, Scopus, and Web of Science databases. The included studies varied widely as they were performed in diverse countries and cultures, which limits the generalizability of these findings to the population of interest. The most frequent limitations of the included studies were related to generalizability and risk of bias. Many were single-center trials with relatively small and homogenous samples, which limits the applicability of findings to broader intensive care unit populations and increases the risk of overestimating treatment effects. Another common issue was inadequate blinding and allocation concealment, raising concerns about performance and detection bias. Additionally, subjective outcome assessments such as self-reported sleep quality instead of objective measures like polysomnography were often used, which may have introduced measurement bias. Finally, environmental factors, specifically unavoidable light and noise exposure in ICUs, and variable adherence to interventions further constrained the reliability and generalizability of the results.

### Implications for practice and further research

Creating a sleep-friendly environment, reducing noise and light levels, and educating staff about the importance of sleep hygiene could further enhance patient outcomes. In terms of research, there remains a need for well-designed, large-scale, multicenter randomized controlled trials to establish the effectiveness of a broader range of sleep-oriented interventions and to understand their long-term impact on delirium prevention. Future studies should also explore the combined effects of multi-component interventions, including environmental modifications, staff education, and individualized patient-centered approaches. Evaluating patient experiences and outcomes beyond the ICU setting may provide additional insights into sustained benefits and best practices for delirium prevention.

### CONCLUSION

This systematic review underscores the promise of sleep-focused, non-pharmacological interventions, especially earplugs and eye masks, in preventing delirium in ICU patients. Although several high-quality studies showed these interventions significantly lowered delirium incidence, some trials found no significant effect, highlighting variability in outcomes. Nevertheless, the overall evidence suggests that simple, safe environmental adjustments and complementary therapies can effectively promote sleep and reduce delirium risk. Considering delirium's complex causes and the drawbacks of medication (39), incorporating non-pharmacological strategies into standard ICU care seems both feasible and advantageous. Future large-scale, well-designed randomized controlled trials are crucial to build stronger evidence and refine clinical practice guidelines.

## Figures and Tables

**Table:** 

Obanor, et al /2021/Texas, USA (18)	Patients admitted to surgical ICU N = 87	N = 44 44 female Mean age: 50.7 (8.7) Earplugs and eye masks	N = 43 43 female Mean age: 51.4 (9.3) Usual routine	Sleep quality, frequency of delirium	Richards-Campbell Sleep Questionnaire	Confusion Assessment Method for the ICU	The average total RCSQ score was significantly higher in the intervention group (*P* = 0.0007). There was no difference in the rate of delirium between the groups, as no patients in either group had positive Confusion Assessment Method scores.	Using earplugs and eye masks improved sleep quality effectively.	9
Shorofi, et al /2024/Iran (24)	Patients undergoing coronary artery bypass grafting in cardiac ICUs N = 114	N = 57 37 male and 20 female Mean age: 58.75 (10.08) Eye masks and earplugs: 9-10 pm until 6-7 am	N = 57 41 male and 16 female Mean age: 60.71 (10.10) Usual routine	Delirium severity and sleep quality	Verran and Snyder-Halpern Sleep Scale	Confusion Assessment Method for the intensive care unit and Neelon and Champagne Confusion Scale	The groups significantly differed in the mean delirium severity score on the second, third, and fourth postoperative day (*P* < 0.001). They also significantly differed in the sleep quality domains: sleep disturbance, sleep effectiveness, and sleep supplementation across the three intervention days (*P* < 0.001).	The use of eye masks and earplugs positively affected sleep quality domains and delirium severity.	11

## References

[1] Al-Hoodar RK Lazarus ER Al Omari O Al Zaabi O Incidence, associated factors, and outcome of delirium among patients admitted to ICUs in Oman. Crit Care Res Pract 2022 2022 1 8 10.1155/2022/4692483 36245554 PMC9553487

[2] Salluh JIF Wang H Schneider EB Nagaraja N Yenokyan G Damluji A Outcome of delirium in critically ill patients: systematic review and meta-analysis. BMJ 2015 350 h2538 10.1136/bmj.h2538 26041151 PMC4454920

[3] Watt CL Momoli F Ansari MT Sikora L Bush SH Hosie A The incidence and prevalence of delirium across palliative care settings: a systematic review. Palliat Med 2019 33 865 77 10.1177/0269216319854944 31184538 PMC6691600

[4] Lange S Mędrzycka-Dąbrowska W Friganovic A Oomen B Krupa S Non-pharmacological nursing interventions to prevent delirium in ICU patients-an umbrella review with implications for evidence-based practice. J Pers Med 2022 12 760 10.3390/jpm12050760 35629183 PMC9143487

[5] Afzal MS Atunde FJ Yousaf RA Pharmacologic management of intensive care unit delirium and the impact on the duration of delirium, length of intensive care unit stay and 30-day mortality: a network meta-analysis of randomized-control trials. Cureus 2023 15 1 10 10.7759/cureus.35843 37033562 PMC10076164

[6] Mart MF Roberson SW Salas B Pandharipande PP Ely EW Prevention and management of delirium in the intensive care unit. Semin Respir Crit Care Med 2021 42 112 26 10.1055/s-0040-1710572 32746469 PMC7855536

[7] Fiest KM Soo A Hee Lee C Niven DJ Ely EW Doig CJ Long-term outcomes in ICU patients with delirium: a population-based cohort study. Am J Respir Crit Care Med 2021 204 412 20 10.1164/rccm.202002-0320OC 33823122 PMC8480248

[8] Irwin SA Pirrello RD Hirst JM Buckholz GT Clarifying delirium management: practical, evidenced-based, expert recommendations for clinical practice. J Palliat Med 2013 16 423 35 10.1089/jpm.2012.0319 23480299 PMC3612281

[9] Barr J Fraser GL Puntillo K Ely EW American College of Critical Care Medicine Clinical practice guidelines for the management of pain, agitation, and delirium in adult patients in the intensive care unit. Crit Care Med 2013 41 263 306 10.1097/CCM.0b013e3182783b72 23269131

[10] NICE. Delirium: prevention, diagnosis and management in hospital and long-term care clinical guideline [CG103]. NICE; 2010. Updated 18/01/2023.

[11] Taylor C Peakman G Mackinnon L Mohamadzade N Han W Mackie L Improving delirium assessments in acute senior health: a quality improvement project for care of the elderly. BMC Geriatr 2024 24 781 10.1186/s12877-024-05273-x 39322946 PMC11423504

[12] Neufeld KJ Yue J Robinson TN Inouye SK Antipsychotic medication for prevention and treatment of delirium in hospitalized adults: a systematic review and meta-analysis. J Am Geriatr Soc 2016 64 705 14 10.1111/jgs.14076 27004732 PMC4840067

[13] Kamdar BB Martin JL Needham DM Promoting sleep to improve delirium in the ICU. Crit Care Med 2016 44 2290 1 10.1097/CCM.0000000000001982 27858818 PMC5599108

[14] Kamdar BB Knauert MP Jones SF Perceptions and practices regarding sleep in the intensive care unit: a survey of 1,223 critical care providers. Ann Am Thorac Soc 2016 13 1370 7 10.1513/AnnalsATS.201601-087OC 27104770 PMC5021080

[15] Patel J Baldwin J Bunting P Laha S The effect of a multicomponent multidisciplinary bundle of interventions on sleep and delirium in medical and surgical intensive care patients. Anaesthesia 2014 69 540 9 10.1111/anae.12638 24813132

[16] Pisani MA D’Ambrosio C Sleep and delirium in adults who are critically ill: a contemporary review. Chest 2020 157 977 84 10.1016/j.chest.2019.12.003 31874132

[17] Arttawejkul P Reutrakul S Muntham D Chirakalwasan N Effect of nighttime earplugs and eye masks on sleep quality in intensive care unit patients. Indian J Crit Care Med 2020 24 6 10 10.5005/jp-journals-10071-23321 32148342 PMC7050172

[18] Obanor OO McBroom MM Elia JM Ahmed F Sasaki JD Murphy KM The impact of earplugs and eye masks on sleep quality in surgical ICU patients at risk for frequent awakenings. Crit Care Med 2021 49 822 32 10.1097/CCM.0000000000005031 33870919

[19] Akpinar RB Aksoy M Kant E Effect of earplug/eye mask on sleep and delirium in intensive care patients. Nurs Crit Care 2022 27 537 45 10.1111/nicc.12741 35021263

[20] Faustino TN Suzart NA dos Santos Rabelo RN Santos JL Batista GS de Freitas YS Effectiveness of combined non-pharmacological interventions in the prevention of delirium in critically ill patients: a randomized clinical trial. J Crit Care 2022 68 114 20 10.1016/j.jcrc.2021.12.015 34999377

[21] Fazlollah A Darzi HB Heidaranlu E Moradian ST The effect of foot reflexology massage on delirium and sleep quality following cardiac surgery: a randomized clinical trial. Complement Ther Med 2021 60 1 7 10.1016/j.ctim.2021.102738 34029674

[22] Kiliç G Kav S Effect of using eye masks and earplugs in preventing delirium in intensive care patients: a single-blinded, randomized, controlled trial. Nurs Crit Care 2023 28 698 708 10.1111/nicc.12901 37138379

[23] Leong RW Davies LJ Fook-Chong S Ng SY Lee YL Effect of the use of earplugs and eye masks on the quality of sleep after major abdominal surgery: a randomised controlled trial. Anaesthesia 2021 76 1482 91 10.1111/anae.15468 33881774

[24] Shorofi SA Dadashian P Arbon P Moosazadeh M The efficacy of earplugs and eye masks for delirium severity and sleep quality in patients undergoing coronary artery bypass grafting in cardiac intensive care units: a single-blind, randomised controlled trial. Aust Crit Care 2024 37 74 83 10.1016/j.aucc.2023.08.003 37802695

[25] Joanna Briggs Institute. Critical Appraisal Tools for use in JBI Systematic Reviews Checklist for Randomized Controlled Trials. Available from: *https://jbi.global/sites/default/files/2019-05/JBI_RCTs_Appraisal_tool**2017**_**0.pdf**.* Accessed: August 24, 2025.

[26] Nahcivan N Seçginli S How to evaluate the methodological quality of quantitative studies included in a systematic review? Turkiye Klinikleri J Public Health Nurs-Special Topics. 2017 3 10 9

[27] Tonna JE Dalton A Presson AP The effect of a quality improvement intervention on sleep and delirium in critically ill patients in a surgical ICU. Chest 2021 160 899 908 10.1016/j.chest.2021.03.030 33773988 PMC8448998

[28] Teng J Qin H Guo W Liu J Sun J Zhang Z Effectiveness of sleep interventions to reduce delirium in critically ill patients: a systematic review and meta-analysis. J Crit Care 2023 78 1 11 10.1016/j.jcrc.2023.154342 37302381

[29] Devlin JW Skrobik Y Gélinas C Needham DM Slooter AJ Pandharipande PP Clinical practice guidelines for the prevention and management of pain, agitation/sedation, delirium, immobility, and sleep disruption in adult patients in the ICU. Crit Care Med 2018 46 e825 73 10.1097/CCM.0000000000003299 30113379

[30] Thrane SE Hsieh K Donahue P Tan A Exline MC Balas MC Could complementary health approaches improve the symptom experience and outcomes of critically ill adults? A systematic review of randomized controlled trials. Complement Ther Med 2019 47 102166 10.1016/j.ctim.2019.07.025 31780011

[31] Karabulut N Aktas YY Nursing management of delirium in the postanesthesia care unit and intensive care unit. J Perianesth Nurs 2016 31 397 405 10.1016/j.jopan.2014.10.006 27667346

[32] Litton E Carnegie V Elliott R Webb SA The efficacy of earplugs as a sleep hygiene strategy for reducing delirium in the ICU: a systematic review and meta-analysis. Crit Care Med 2016 44 992 9 10.1097/CCM.0000000000001557 26741578

[33] Van Rompaey B Elseviers M Van Drom W Fromont V Jorens P The effect of earplugs during the night on the onset of delirium and sleep perception: a randomized controlled trial in intensive care patients. Crit Care 2012 16 1 11 10.1186/cc11330 22559080 PMC3580615

[34] Patel J Baldwin J Bunting P Laha S The effect of a multicomponent multidisciplinary bundle of interventions on sleep and delirium in medical and surgical intensive care patients. Anaesthesia 2014 69 540 9 10.1111/anae.12638 24813132

[35] Chen TJ Traynor V Wang AY Shih CY Tu MC Chuang CH Comparative eeffectiveness of non-pharmacological interventions for preventing delirium in critically ill adults: A systematic review and network meta-analysis. Int J Nurs Stud 2022 131 104239 10.1016/j.ijnurstu.2022.104239 35468538

[36] Kang J Lee M Ko H Kim S Yun S Jeong YChY Effect of nonpharmacological interventions for the prevention of delirium in the intensive care unit: A systematic review and meta-analysis. J Crit Care 2018 48 372 84 10.1016/j.jcrc.2018.09.032 30300863

[37] Deng LX Cao L Zhang LN Peng XB Zhang L Non-pharmacological interventions to reduce the incidence and duration of delirium in critically ill patients: A systematic review and network meta-analysis. J Crit Care 2020 60 241 8 10.1016/j.jcrc.2020.08.019 32919363

[38] Grafton-Clarke C Grace L Roberts N Harky A Can postoperative massage therapy reduce pain and anxiety in cardiac surgery patients? Interact Cardiovasc Thorac Surg 2019 28 716 21 10.1093/icvts/ivy310 30508186

[39] Li J Fan Y Luo R Yin N Wang Y Jing J The impact of non-pharmacological sleep interventions on delirium prevention and sleep improvement in postoperative ICU patients: A systematic review and network meta-analysis. Intensive Crit Care Nurs 2025 87 103925 10.1016/j.iccn.2024.103925 39709722

